# Network Approach to Source Attribution of *Salmonella enterica* Serovar Typhimurium and Its Monophasic Variant

**DOI:** 10.3389/fmicb.2020.01205

**Published:** 2020-06-16

**Authors:** Alessandra Merlotti, Gerardo Manfreda, Nanna Munck, Tine Hald, Eva Litrup, Eva Møller Nielsen, Daniel Remondini, Frédérique Pasquali

**Affiliations:** ^1^Department of Physics and Astronomy, Alma Mater Studiorum – University of Bologna, Bologna, Italy; ^2^Department of Agricultural and Food Sciences, Alma Mater Studiorum – University of Bologna, Bologna, Italy; ^3^National Food Institute, Technical University of Denmark, Copenhagen, Denmark; ^4^Statens Serum Institute, Copenhagen, Denmark

**Keywords:** source attribution, *Salmonella enterica*, network analysis, SNP distance, multi locus sequence typing, whole genome multi locus sequence typing

## Abstract

*Salmonella enterica* subspecies enterica serovar Typhimurium and its monophasic variant are among the most common *Salmonella* serovars associated with human salmonellosis each year. Related infections are often due to consumption of contaminated meat of pig, cattle, and poultry origin. In order to evaluate novel microbial subtyping methods for source attribution, an approach based on weighted networks was applied on 141 human and 210 food and animal isolates of pigs, broilers, layers, ducks, and cattle collected in Denmark from 2013 to 2014. A whole-genome SNP calling was performed along with cgMLST and wgMLST. Based on these genomic input data, pairwise distance matrices were built and used as input for construction of a weighted network where nodes represent genomes and links to distances. Analyzing food and animal Typhimurium genomes, the coherence of source clustering ranged from 89 to 90% for animal source, from 84 to 85% for country, and from 63 to 65% for year of isolation and was equal to 82% for serotype, suggesting animal source as the first driver of clustering formation. Adding human isolate genomes to the network, a percentage between 93.6 and 97.2% clustered with the existing component and only a percentage between 2.8 and 6.4% appeared as not attributed to any animal sources. The majority of human genomes were attributed to pigs with probabilities ranging from 83.9 to 84.5%, followed by broilers, ducks, cattle, and layers in descending order. In conclusion, a weighted network approach based on pairwise SNPs, cgMLST, and wgMLST matrices showed promising results for source attribution studies.

## Introduction

*Salmonella enterica* subspecies enterica serovar Typhimurium and its monophasic variant (STm) are among the top three serovars in confirmed human cases of salmonellosis each year in Europe (EFSA and ECDC, 2018). Although in 2017, in comparison to the previous year, the percentage of confirmed human cases associated with the monophasic variant was similar, specific concern arose in recent years due to the emergence of outbreaks worldwide since 2006 (Mossong et al., 2006; Bone et al., 2010; Raguenaud et al., 2012; Barco et al., 2014; Cito et al., 2016; de Frutos et al., 2018; Dewey-Mattia et al., 2018).

*Salmonella typhimurium* can infect humans from different sources. However, in 2017 the most reported matrices were broiler, pig, turkey, and layers in decreasing order for *S. typhimurium*, and pig and broilers for its monophasic variant accounting for 49.7 and 35.3%, respectively (EFSA and ECDC, 2018).

Attributing cases of Salmonellosis to specific sources is crucial to identify and prioritize targeted interventions in the food chain, as well as to evaluate the effectiveness of each intervention. Many methods have been developed to estimate the relative contribution of different food sources to human foodborne diseases worldwide, including microbial subtyping, comparative exposure assessment, epidemiological analysis of sporadic cases, analysis of data from outbreak investigations, and expert elicitation (Pires et al., 2009, 2014). Each of these approaches has strengths and limitations, and the usefulness of each depends on the public health questions being addressed (Pires et al., 2014). Usually, source attribution studies are conducted by using frequency-matching models like the Dutch and Danish models based on phenotyping data (serotyping, phage-typing, and antimicrobial resistance profiling) (Pires et al., 2009, 2014; Mughini-Gras et al., 2018).

A different approach to source attribution might be envisaged based on the theory of weighed networks. There are many examples of network modeling applications in different fields, such as computer and information sciences, social sciences, and biology (Newman, 2010). In biomedical fields, networks are powerful tools to perform characterization, classification, and ranking of interacting elements in a complex biological system (Bersanelli et al., 2016). Specifically, for source attribution, pairwise distance matrices can be interpreted as fully connected networks where nodes correspond to bacterial isolates and links to genetic distances (i.e., number of different nucleotides along DNA sequences). The weaker the link, the higher the genetic distance within two isolates. The aim is to extract network communities corresponding to different animal sources, whether they are separate subnetworks (disconnected components, as in our case) or node clusters obtained by exploiting the plethora of community detection algorithms known from network theory literature (Fortunato and Hric, 2016). The probability of a human isolate to be associated with a specific animal source is computed as a function of the number of links the human isolate has with other isolates of specific animal sources.

The network approach is useful also in investigating which structural features of a data set play a fundamental role in determining the internal coherence of clusters. Apart from animal sources, the country of origin of imported food samples and year of collection might impact the clustering formation.

Any kind of subtyping data can be used to establish distances between nodes: phenotypic data such as serotyping, molecular data such as 7-gene MLST, or genomic data such as single-nucleotide polymorphism (SNP) calling and core-genome or whole-genome multi-locus sequence typing (cgMLST and wgMLST).

Recently, phenotyping and molecular data are increasingly replaced by genomic data with high discriminatory power required to distinguish strains of a monomorphic serovar such as the *S. typhimurium* monophasic variant (Gymoese et al., 2017; Morganti et al., 2018; Palma et al., 2018). In particular, among molecular subtyping methods, 7-gene MLST has been described as phylogenetically able to rank up *Salmonella* to the species level and even, occasionally, at the subspecies and serotype level but not discriminatory enough for the purpose of source tracking (Leekitcharoenphon et al., 2012; Ferrari et al., 2017).

Whole-genome sequencing (WGS) showed up as an impressive one-serve-all approach providing a great amount of data, which not only enables subtyping at high resolution but also provides valuable additional information for further characterization of the isolate (i.e., virulence, antimicrobial resistance). A retrospective study of *S. typhimurium* and its monophasic variant showed that SNP-based WGS analysis was suitable for subtyping these two serovars. Specifically, a core-SNP analysis was found valuable for *S. typhimurium*, whereas a wider approach such as a whole-genome-SNP analysis was suggested as more suitable for the tight genetic group of its monophasic variant (Gymoese et al., 2017).

However, SNP analysis shows a number of drawbacks linked to the lack of standardization of the workflow. The choice of the reference genome, the quality of sequences, and the bioinformatics tool chosen for SNP calling may have an effect on the type and number of SNPs (Gymoese et al., 2017). Alternatively, for an inter-laboratory comparison, cgMLST and wgMLST are gene-by-gene approaches with fixed allele schemes widely applied in surveillance. While the traditional PCR-based MLST is based on a fixed scheme of 7 alleles/genes, cgMLST and wgMLST schemes include thousands of genes dramatically increasing the discriminatory power of these WGS-based subtyping methods.

Since different food sources can be attributed to human Salmonella infections, there is a specific need in developing accurate estimates of the relative contributions of different exposures to the total number of human cases. This analysis is relevant for optimal priority-setting for control and intervention strategies. With the change to whole-genome sequencing, there is a need to develop new methods for source attribution based on sequencing data. Munck et al. (2020) have recently presented four European datasets collected in Denmark, Germany, United Kingdom, and France and including sequenced genomes of *S. typhimurium* and its monophasic variants isolated from human, food, animal, and the environment during the time frame 2013–2016. The objective of the Danish, United Kingdom, and German datasets was to attribute the human salmonellosis cases to animal reservoirs, while the objective of the French dataset was to attribute the environmental isolates to animal reservoirs in order to investigate the environmental contamination (Munck et al., 2020).

The aim of the present study was to evaluate the potential application of the weighted network approach to source attribution using whole-genome data. We considered the Danish dataset previously described, comprising 141 human and 210 food and animal isolates of pig, broiler, layer, duck, and cattle collected from 2013 to 2014. We applied the network-based method to different input data, namely, distance matrices obtained from SNP, cgMLST, and wgMLST data. We did not perform any outbreak detection analysis, since we had no data available for this aim.

## Materials and Methods

### Dataset

The dataset was previously described and collected with the purpose of estimating the sources of human infections in Denmark. The dataset includes 351 *S. typhimurium* and its monophasic variant isolates: 210 isolates collected from food of animal origin (pigs, broilers, ducks, layers, and cattle) and 141 isolates of human origin. All human isolates were from Denmark. Food isolates were from Denmark as well as imported to Denmark from four different countries: Germany, Ireland, United Kingdom, and others). All isolates were collected in 2013 and 2014 (Tables 1–3; Munck et al., 2020).

**TABLE 1:** Data set composition according to primary source and sampling year.

	**2013**	**2014**	**Total**
Broilers	13	21	34
Pigs	104	55	159
Ducks	0	11	11
Cattle	1	1	2
Layers	3	1	4
Human	29	112	141
Total	150	201	351

**TABLE 2:** Data set composition according to primary source and serovar.

	**Monophasic**	**Typhimurium**	**Total**
Broilers	16	18	34
Pigs	84	75	159
Ducks	1	10	11
Cattle	1	1	2
Layers	0	4	4
Human	68	73	141
Total	170	181	351

**TABLE 3:** Data set composition according to primary source and country of sample origin.

	**Denmark**	**Imported to Denmark**				**Total**
		**Germany**	**Others**	**Ireland**	**United Kingdom**	
Broilers	34	0	0	0	0	34
Pigs	125	32	0	1	1	159
Ducks	0	0	11	0	0	11
Cattle	1	0	1	0	0	2
Layers	4	0	0	0	0	4
Human	141	0	0	0	0	141
Total	305	32	12	1	1	351

### Genomic Subtyping

#### SNP Calling

Single-nucleotide polymorphism calling was performed using the CSI Phylogeny pipeline available as a webtool through the Center for Genomic Epidemiology^1^ as previously reported. The following options were used: Select min. depth at SNP positions: 10x. Select min. relative depth at SNP positions: 10%. Select minimum distance between SNPs (prune): 10. Select min. SNP quality: 30. Select min. read mapping quality: 25. Select min. Z-score: 1.96.

#### cgMLST and wgMLST

Core-genome multi-locus sequence typing (cgMLST) and whole-genome multi-locus sequence typing (wgMLST) analyses were performed in BioNumerics version 7.6 (Applied Maths, Sint-Martens-Latem, Belgium). cgMLST were obtained using the Enterobase scheme (Alikhan et al., 2018) in BioNumerics, and wgMLST was performed based on the scheme developed by Applied Maths. The core-genome scheme of *Salmonella* consists of 3,002 loci, and the whole-genome scheme consists of 21,065 loci with one single locus having several allele variations. Isolates were considered for analysis when presenting a core-genome coverage higher than 95% (corresponding to 2,852 loci) and a detection of mixed sequence alleles lower than 50 alleles.

### Weighted Network

**FIGURE 1:**
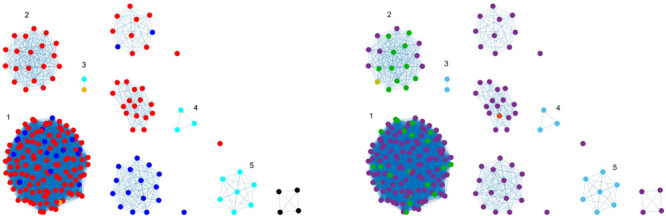
Clustering results (force-directed graph drawing algorithm) obtained by SNP distance matrix, where different node colors represent different sources **(left)** and different countries of origin **(right)**. Legend for left-hand figure: pigs, red; broilers, blue; cattle, yellow; ducks, cyan; layers, black. Legend for right-hand figure: purple, Denmark; green, Germany; light blue, others; orange, United Kingdom; yellow, Ireland.

**FIGURE 2:**
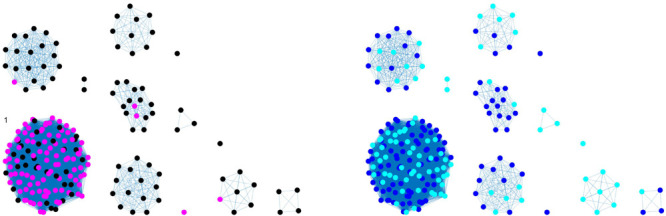
Clustering results (force-directed graph drawing algorithm) obtained by SNP distance matrix, where different node colors represent different serotypes **(left)** and different sampling years **(right)**. Legend for left-hand figure: pink, monophasic; black, Typhimurium. Legend for right-hand figure: 2013, blue; 2014, cyan.

Each matrix was represented as a network, where nodes correspond to isolates, and links are a function of pairwise distance *d*_*ij*_ calculated as the number of different nucleotides or number of different alleles between two isolate DNA sequences *i* and *j*. In order to find an association between distances and animal sources, the following assumption was made: genomes coming from the same source should show smaller distance values. Therefore, a fully connected weighted network W (in which the weight *w_*ij*_* = *1/d_*ij*_* was assigned to each link between samples *i* and *j*) was built. Subsequently, a threshold was applied to the constructed weight matrices in order to remove weaker links (associated with larger genetic distances). In the resulting binarized network, nodes were linked by an edge only if their weight was greater than a given threshold value, and source clusters were identified as the disconnected components (i.e., groups of nodes with links within each other but not with the nodes of other components; see Figures 1, 2, 4, 5) obtained by the thresholding procedure. The threshold value was chosen in order to maximize internal coherence of clusters and minimize the number of isolated nodes. Precisely, the best threshold value *t* was found through a 70/30 cross-validation procedure applied on animal source data, aiming to maximize the following score function on distance matrices:

$$s\,c\,o\,r\,e=\left(1-\frac{N_{I\,S\,O}}{N_{T\,O\,T}}\right)\,C\,S\,C$$

where *N*_*TOT*_ represents the total number of nodes in the network, *N*_*ISO*_ represents the number of isolated nodes (i.e., not forming any link with other nodes), and *CSC* represents the coherent source clustering, the parameter that estimates algorithm clustering performance, computed as follows:

$$C\,S\,C=\frac{\sum_{i=1}^{N_{c}}T\,P_{i}}{\sum_{i=1}^{N_{c}}T_{i}}\,100$$

where *TP*_*i*_ represents the number of true positives inside the *i*th cluster (i.e., the isolates from the same source found in the same cluster) and *T*_*i*_ the total number of nodes inside the *i*th cluster.

Specifically, the 70/30 cross-validation procedure is structured as follows: 70% of the animal origin samples were randomly selected in order to build a network (training set) on which the best threshold value *t* was computed by maximizing the score function and then applied to the network constructed with the remaining 30% samples (test set), in which the clustering performance was evaluated. This procedure was repeated 100 times, with different random 70/30 data set subdivisions, and the most frequent value was selected as the global best threshold *t* for source clustering. It was then applied to the distance matrix obtained from the whole dataset, including both human and animal Typhimurium genomes, so that human samples could be attributed to a putative source according to the following rule:

$$\max=\frac{l_{j}}{L}\;j=1,\ldots .,n$$

where *n* represents the number of different animal origin sources, *l*_*j*_ represents the number of links between a single human isolate *h* and all nodes belonging to the *j*th animal source and *L* represents the number of all neighbors of animal origin of the human sample. The ratio *l_*j*_/L* can be considered as the best estimate of the probability that a human isolate *h* is attributed to the *j*th animal origin source, given the available dataset.

Graphical representations of networks (Figures 1, 2, 4, 5) were generated using MathWorks Matlab “plot” function with a force-directed graph layout (Fruchterman and Reingold, 1991).

## Results

### Genomic Subtyping

#### SNP Calling

Single-nucleotide polymorphism-based phylogenetic analysis found 15,831 SNPs. In general, the isolates were clustered according to *Salmonella* serogroup and ST-type (see Supplementary Table S2). In addition, human isolates were intermixed with the potential sources. Some of the imported duck and domestic layer isolates clustered together and showed higher similarity with 385 (min: 1, max: 742) average SNP difference for imported ducks and 340 (min: 1, max: 631) for domestic layers whereas the domestic and imported pig isolates were highly diverse and scattered around the tree (Munck et al., 2020). The isolates from domestic broilers formed two groups described by serotype. An outgroup with on average 1549 (min: 1,178, max: 1,770) pairwise SNP difference is linked to four human ST36 isolates. One of these cases was travel related, and the remaining had unknown travel history.

Single-nucleotide polymorphism difference between human and animal isolates showed higher values, going from an average of 648 (min: 0, max: 8815) between human and broilers, to an average of 1251 (min: 166, max: 8965) between human and imported ducks (see Supplementary Table S3).

#### cgMLST and wgMLST

The cgMLST genome analysis detected all 3002 loci found in the scheme in at least one of the genomes. 469 loci had identical alleles in all genomes analyzed, but looking at only 99% of the genomes, the number of identical loci goes up to 1141 of the 3002 in the scheme. The wgMLST analysis detected a total of 6433 loci present in at least one genome. The number of loci with identical alleles detected in all genomes was 1911, whereas this number increases to 2859 loci when looking at 99% of the genomes. The four strains of Typhimurium belonging to another clonal complex (all are ST36) are responsible for much of the polymorphism detected in the dataset.

### Weighted Network

**FIGURE 3:**
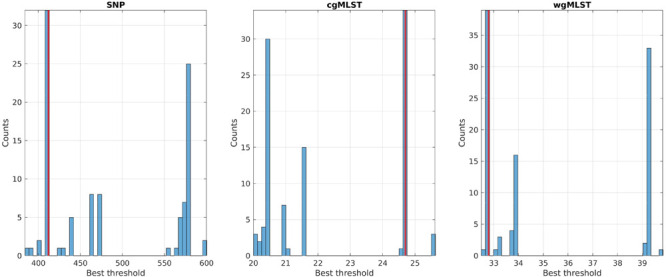
Best threshold values obtained by the 70/30 cross-validation procedure on the training sets of SNP, cgMLST, and wgMLST distance matrices. The red line corresponds to the global best threshold value used for source clustering (Figure 4) and source attribution (Figure 5).

**FIGURE 4:**
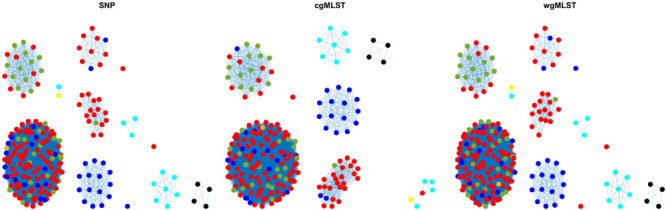
Source clustering results (force-directed graph drawing algorithm) obtained by SNP, cgMLST, and wgMLST distance matrices. Legend: pigs from Denmark, red; import pigs, green; broilers, blue; cattle from Denmark, darker yellow; import cattle, lighter yellow; ducks, cyan; layers, black.

**FIGURE 5:**
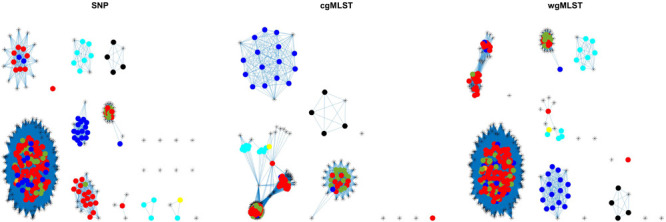
Source attribution results (force-directed graph drawing algorithm) obtained by SNP, cgMLST, and wgMLST distance matrices. Legend: pigs from Denmark, red; import pigs, green; broilers, blue; cattle from Denmark, darker yellow; import cattle, lighter yellow; ducks, cyan; layers, black; humans, asterisk.

Best threshold values, obtained with the cross-validation procedure, were 412, 24.7, and 32.79 for SNP, cgMLST, and wgMLST matrices, respectively, since they maximized the score function and corresponded to the most probable values obtained from 100 cross-validation runs (Figure 3). In particular, the structure of the disjoint connected components shown in Figures 1, 2, 4, 5 could be achieved by considering only pairwise genomic distances lower than these threshold values.

The method reaches 90% of coherent source clustering for animal source on SNP and wgMLST matrices and 89% on the cgMLST matrix, showing that animal source type is the main factor driving cluster formation, followed by country of origin, serotype, and sampling year (Table 4). Although the overall algorithm performance is good, broilers and cattle represent the most difficult sources to detect: 18 out of 34 among the former as well as 1 out of 2 of the latter are classified as pigs (see Table 5 for SNP and wgMLST distance matrices and Supplementary Table S1 for cgMLST distance matrix), being included in the same network component. Figures 1, 2, on the left-hand sides, show that most of the confusion between broilers and pigs arises from cluster 1, which is mainly composed of isolates of monophasic variant: their peculiar low variability at the genomic level could be the reason for encountered difficulties in distinguishing the two different sources (Mastrorilli et al., 2018).

**TABLE 4:** Coherent source clustering (CSC) for SNP, cgMLST, and wgMLST distance matrix, computed on animal origin isolates, according to the following parameters: animal source, serotype, country of origin, and sampling year.

**CSC**	**SNP**	**cgMLST**	**wgMLST**
Animal source	90%	89%	90%
Country of origin	85%	84%	85%
Serotype	82%	82%	82%
Sampling year	65%	63%	65%

**TABLE 5:** Confusion matrix obtained from source clustering results that are the same for SNP and wgMLST distance matrices.

		**PREDICTED**				
		**Broilers**	**Cattle**	**Ducks**	**Layers**	**Pigs**
TRUE	Broilers	16	0	0	0	18
	Cattle	0	0	1	0	1
	Ducks	0	0	10	0	0
	Layers	0	0	0	4	0
	Pigs	0	0	0	0	159

In terms of cluster structure, most of the subnetworks are composed of the same type of animal source except for cluster 1, where pig and broiler isolates are mixed together with one of the cattle samples (Figure 1, left-hand side). Regarding country of origin of imported food samples, regionality affects cluster formation since most of import isolates tend to group apart from those from Denmark, as confirmed by cluster 2, mainly composed of pig isolates from Germany and by clusters 3, 4, and 5, mainly composed of import ducks and cattle isolates (Figure 1, right-hand side). Another relevant parameter for cluster formation is serotype, as confirmed by subnetwork composition, since a clear separation between genomes of Typhimurium and its monophasic variant was observed. In particular, genomes of *S. typhimurium* monophasic variant cluster all together in subnetwork 1, whereas *S. typhimurium* showed a more heterogeneous behavior, especially for pig isolates, which appeared stratified in more than one group (Figure 2, left-hand side). Finally, sampling year had no impact on cluster formation, as confirmed by the high variability in terms of cluster composition (Figure 2, right-hand side).

**FIGURE 6:**
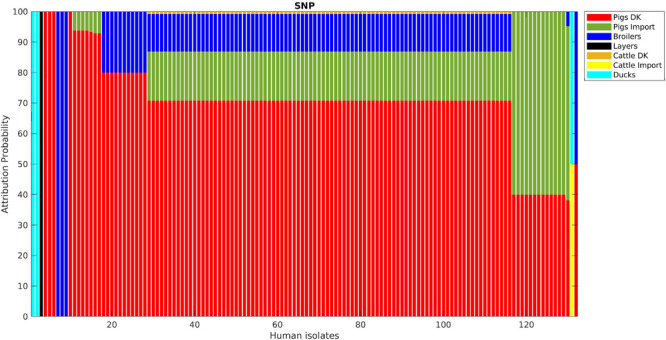
Human isolate probability to originate from each source as determined by source attribution analysis via the network-based approach on the SNP pairwise distance matrix.

**FIGURE 7:**
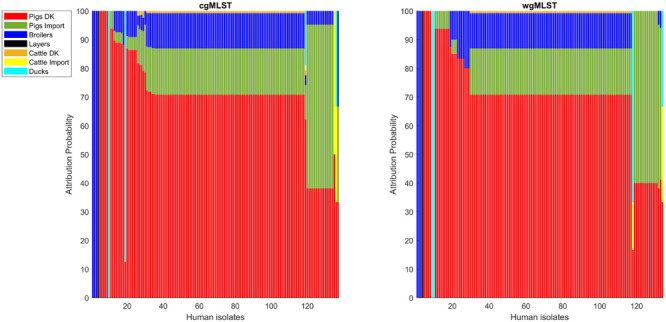
Human isolate probability to originate from each source on the cgMLST pairwise distance matrix **(left)** and on the wgMLST pairwise distance matrix **(right)**.

Adding human isolate genomes to the network (Figure 5), a percentage between 93.6 and 97.2% clustered with the existing animal network components, and only a percentage between 2.8% (from cgMLST distance matrix) and 6.4% (from SNPs distance matrix) appeared as not linked to any animal Typhimurium genome (Table 6). One of those human isolated nodes was travel related (data not shown). The majority of attributable human genomes were associated with pigs with probabilities ranging from 83.9 (SNP matrix) to 84.5% (cgMLST and wgMLST matrices), followed by broilers, ducks, cattle, and layers in descending order (Table 7). We remark that even if the dataset presents a large abundance of pig and broiler samples, we also found human isolates with 100% links toward less abundant animal sources, such as layers and ducks, reflecting the fact that our analysis does not seem heavily affected by such source representation imbalance. Moreover, if we further stratify pigs by distinguishing between import and non-import (Table 8), we can notice that most of the human isolates are associated with non-import pigs, with probabilities ranging from 66.4 (SNP matrix) to 66.7% (cgMLST and wgMLST matrices). The same stratification can be applied also to cattle, but the probability that a human isolate is associated with an import and non-import cattle is almost the same due to the very low number of genomes. Furthermore, links between human samples and animal sources showed high specificity in all the three networks (SNP, cgMLST, and wgMLST) as confirmed in Figures 6, 7, making the putative originating animal source clearly attributable.

**TABLE 6:** Percentage of attributed and not attributed human isolates.

**Human isolates**	**SNP**	**cgMLST**	**wgMLST**
Attributed	93.6%	97.2%	95.0%
Not attributed	6.4%	2.8%	5.0%
			

**TABLE 7:** Mean probability (expressed in percentage) of a human isolate to be attributed to a source, together with confidence intervals, calculated for each of the considered pairwise distance matrices (SNP, cgMLST, and wgMLST).

**Source**	**SNP**	**cgMLST**	**wgMLST**
Broilers	12.5(10.0–15.1)	11.8(9.4–14.2)	11.7(9.2–14.1)
Cattle	0.9(0.1–1.6)	1.2(0.3–2.0)	0.9(0.3–1.4)
Ducks	1.9(0–4.1)	1.8(0–3.7)	2.2(0–4.6)
Layers	0.7(0–2.2)	0.7(0–2.2)	0.7(0–2.2)
Pigs	83.9(80.3–87.5)	84.5(81.3–87.8)	84.5(80.1–88.0)

**TABLE 8:** Mean probability (expressed in percentage) of a human isolate to be attributed to a source, together with confidence intervals, calculated for each of the considered pairwise distance matrices (SNP, cgMLST, and wgMLST).

**Source**	**SNP**	**cgMLST**	**wgMLST**
Broilers	12.5(10.0–15.1)	11.8(9.4–14.1)	11.7(9.2–14.1)
Cattle	0.5(0.4–0.6)	0.6(0.5–0.6)	0.5(0.4–0.6)
Cattle import	0.4(0–1.1)	0.6(0–1.5)	0.4(0–0.9)
Ducks	1.9(0–4.1)	1.7(0–3.7)	2.2(0–4.6)
Layers	0.7(0–2.2)	0.7(0–2.2)	0.7(0–2.2)
Pigs	66.4(62.9–69.9)	66.7(63.3–70.1)	66.7(63.2–70.2)
Pig import	17.5(14.7–20.2)	17.9(15.3–20.4)	17.7(15.0–20.5)

*In this case, we took into account the stratification of pigs and cattle into import and non-import.*

Overall, similar source clustering results (see Figure 4) and human isolate source attribution are obtained for the three different genetic distances (SNP, cgMLST, and wgMLST) suggesting the robustness of results independently of the subtyping method (Figures 6, 7 and Tables 5–8).

## Discussion

In this study, an approach based on weighted networks was used to attribute domestic human *Salmonella* infections that occurred in Denmark from 2013 to 2014 to five different potential animal sources, namely, pigs, broilers, laying hens, cattle, and ducks.

Pigs were by the far most frequent animal source to which human genomes of *S. typhimurium* and its monophasic variant were attributed in this study. This result is not surprising. Excluding traveling, other authors have been describing pigs as the main source of human *Salmonella* infections in Denmark as well as in Southern Europe for two decades with estimated percentages ranging from 15% (Denmark) to 44% (Italy) (Mughini-Gras et al., 2014; Pires et al., 2014). The higher values in the present study are linked to the dataset which exclusively includes serovar *S. typhimurium* and its monophasic variant, historically associated with pig reservoir. In particular, in the period 2013–2014 *S. typhimurium* was the most frequently detected serovar in pigs and pig meat in Europe (EFSA and ECDC, 2015a, b). However, it must be pointed out that, although a shift from broilers to pigs was described over the last 20 years specifically after the implementation of *Salmonella* control programs in poultry (EU Regulation (EC) No. 2160/2003, 2003), a different trend might be observed in the near future. In particular, in 2017 although the main reservoir of human infections associated with the monophasic variant remains pig, for *S. typhimurium* infections, broilers are indicated for the first time as the most reported animal food source in Europe (EFSA and ECDC, 2018).

Several methods have been proposed for source attribution of foodborne diseases (Pires et al., 2014). Regarding the microbial subtyping approaches, the aim is to compare subtypes of isolates from different animal sources to those from humans with the final purpose of estimating the percentage of human *Salmonella* infections attributable to each specific source. This approach is based on the principle of a strong association between subtypes and specific animal reservoirs and requires a representative collection at the point of production of temporally and spatially related isolates from various sources. Differently from the Hald and the Dutch models based on the comparison of Bayesian measures of frequency distributions of *Salmonella* subtypes in animals and humans, this network-based approach is based on a direct comparison of genomic distances among isolates of different reservoirs that provide the weights of network links. This approach allows to verify the association between subtypes and animal reservoirs by evaluating the clustering coherence of reservoirs and more in general the driving force of each parameter (reservoir, country of origin, year distance) in clustering formation, by comparing the obtained clusters with the parameter labels distributed on the nodes. In the present-study animal source was the major driving force of clustering formation, followed by the country of origin of imported food samples and serovar. Year of isolation did not impact significantly, although it must be underlined that the time period was only 2 years. A higher impact of the year of isolation would have been probably observed in the case of longer time frames (i.e., 10 or more years). Results on the impact of the country of origin are in line with the high relatedness of *S. typhimurium* subtypes in isolates of the same geographic area, recently highlighted also in a study describing a geographical segregated genomic clade of the *S. typhimurium* monophasic variant in Italy (Palma et al., 2018).

In principle, the discriminatory power of the subtyping method is of crucial importance in source attribution. The low discriminatory power of phenotypic subtyping, for example, might lead to poor differentiation among isolates of different animal reservoirs impairing the cluster coherence. In accordance with previously papers, results of 7-gene MLST of the dataset of the present study confirm 7-gene MLST not enough discriminatory for the purpose of source tracking. In particular, the majority of the genomes were categorized in only two ST types, namely, ST19 and ST34 (Supplementary Table S2; Leekitcharoenphon et al., 2012; Ferrari et al., 2017).

On the other hand, the high discriminatory power of genomic subtyping might lead to unjustified differentiation with the identification of too many clusters and a higher number of not attributable human isolates (Barco et al., 2013; Pires et al., 2014). Although highly discriminatory, the three genomic subtyping datasets used in the present study as input data (SNP calling, cgMLST, and wgMLST) showed a good discriminatory power in order to maximize cluster coherence and minimize the number of human isolated nodes corresponding to not attributable human genomes. Although this was the case for *S. typhimurium* isolates of the present study, the same might not be true for other bacterial pathogens with higher intraspecies genetic diversity such as *Campylobacter* (Woodcock et al., 2017). Besides discriminatory power, the output of the network analysis revealed that, although wgMLST generally offers higher resolution than cgMLST, source attribution results did not differ significantly, demonstrating the robustness of the approach.

One of the major concerns on all source attribution approaches is the estimate percentages of human infections of unknown sources or not attributable human infections. With the network approach, less than 7% of human genomes were not attributed to any animal source. This value is lower than those previously reported for other microbial subtyping methods for source attribution (Pires et al., 2014). However, along with the model approach, the dataset itself might strongly influence this estimate especially in case the dataset does not fully represent the real temporal and spatial distribution of human and animal subtypes/isolates.

## Conclusion

In conclusion, this is the first report in which a method based on weighted networks is successfully applied to a source attribution research question. The approach provides estimate percentages of human infections associated with *S. typhimurium* and its monophasic variant, to each specific source by comparing genomic weighted distances among genomes of different reservoirs. Results were robust, independently from the genomic data used as input. Further studies need to be performed on different datasets in order to confirm the usefulness and reliability of the weighted network approach on source attribution studies applied to *Salmonella* infections.

## Data Availability Statement

The datasets generated for this study can be found in the European Nucleotide Archive under accession number PRJEB14853.

## Author Contributions

AM performed the analysis. DR designed the analysis and contributed to drafting the manuscript. TH and NM performed SNP calling. EN and EL performed cgMLST and wgMLST. FP supervised and coordinated the entire work, performed literature search, and drafted the manuscript. All authors contributed to data interpretation, manuscript revision, and approved the final version as submitted. GM supervised the analyses and interpreted the results.

## Conflict of Interest

The authors declare that the research was conducted in the absence of any commercial or financial relationships that could be construed as a potential conflict of interest.

## Footnotes

1 www.genomicepidemiology.org
