# Supplementary material for: Network Approach to Source Attribution of Salmonella enterica Serovar Typhimurium and Its Monophasic Variant
Source: Front Microbiol. 2020 Jun 16;11:1205. doi: 10.3389/fmicb.2020.01205 (PMC8335978; doi:10.3389/fmicb.2020.01205)
Supplement: Supplementary file 1 [file Data_Sheet_1.PDF]

Supplementary material

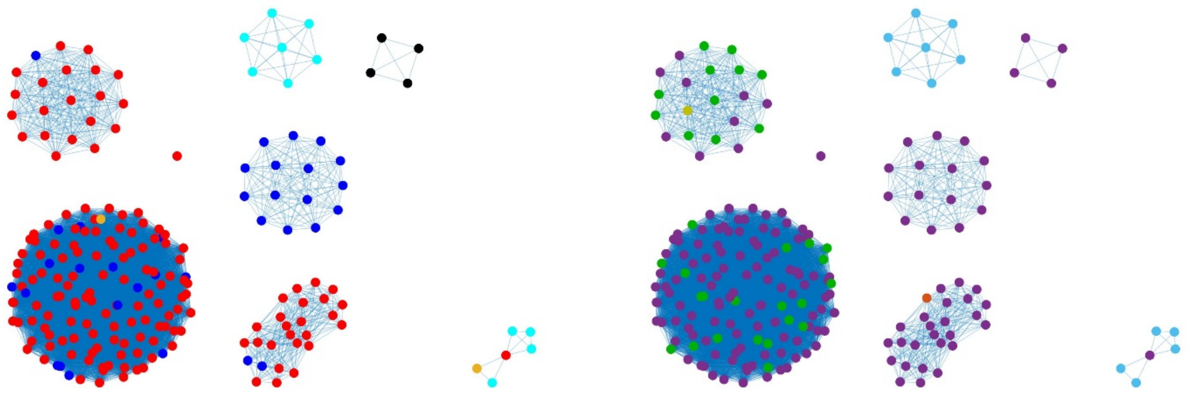

Figure S1: *Clustering results (force-directed graph drawing algorithm) obtained by cgMLST distance matrix, where different node colors represent different sources (left) and different countries of origin (right). Legend for left-hand figure: pigs (red), broilers (blue), cattle (yellow), ducks (cyan), layers (black). Legend for righ-hand figure: purple (Denmark), green (Germany), light blue (others), orange (UK), yellow (Ireland).*

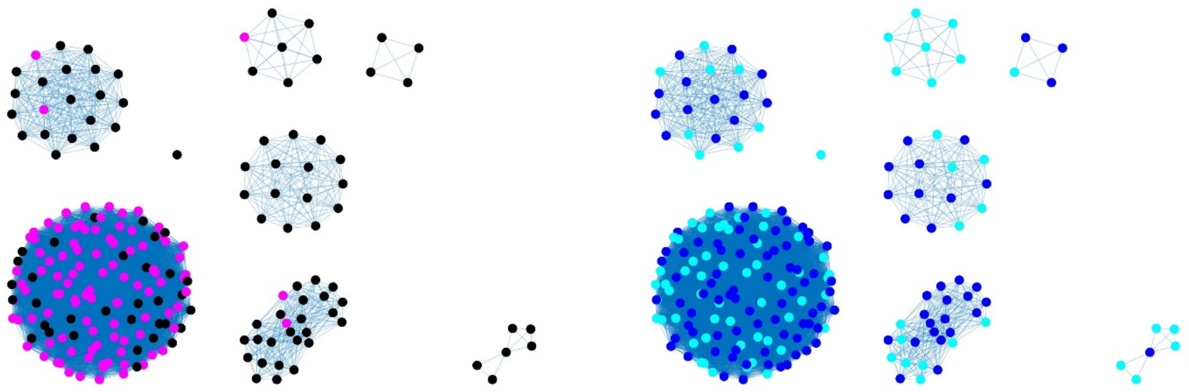

Figure S2: *Clustering results (force-directed graph drawing algorithm) obtained by cgMLST distance matrix, where different node colors represent different serotypes (left) and different sampling years (right). Legend for left-hand figure: pink (Monophasic), black (Typhimurium). Legend for right-hand figure : 2013 (blue), 2014 (cyan).*

Table S1 - Confusion matrix obtained from source clustering results of cgMLST distance matrix.

		PREDICTED				
		Broilers	Cattle	Ducks	Layers	Pigs
TRUE	Broilers	15	0	0	0	19
	Cattle	0	0	1	0	1
	Ducks	0	0	10	0	0
	Layers	0	0	0	4	0
	Pigs	0	0	1	0	158

Table S2 – Number of isolates per source belonging to each of the identified ST types.

	ST19	ST34	ST36	ST376	ST568	ST2212
Broilers	18	16	0	0	0	0
Cattle	1	1	0	0	0	0
Ducks	11	0	0	0	0	0
Layers	4	0	0	0	0	0
Pigs	46	113	0	0	0	0
Human	49	85	4	1	1	1

Table S3 – Mean, minimum and maximum SNP distance value between human isolates and each animal source.

	Mean	Minimum	Maximum
Human-Broilers	648	0	8815
Human-Cattle	880	7	8997
Human-Ducks	1251	166	8965
Human-Layers	776	12	8711
Human-Pigs	1123	3	8939
